# Digital Mahjong-Based Cognitive Engagement Intervention for Older Adults: Randomized Controlled Trial

**DOI:** 10.2196/85339

**Published:** 2026-07-23

**Authors:** Heng-Hsin Tung, Chen-Yuan Kuo, Pei-Lin Lee, Chih-Wen Chang, Kun-Hsien Chou, Katelyn E Chen, Ching-Po Lin, Chih-Kuang Liang, Liang-Kung Chen

**Affiliations:** 1 Department of Nursing National Yang Ming Chiao Tung University Taipei City Taiwan; 2 Center for Healthy Longevity and Aging Sciences National Yang Ming Chiao Tung University Taipei City Taiwan; 3 Tungs’ Taichung Metro Harbor Hospital Taichung City Taiwan; 4 Institute of Neuroscience National Yang Ming Chiao Tung University Taipei City Taiwan; 5 Brain Research Center National Yang Ming Chiao Tung University Taipei City Taiwan; 6 Department of Mechanical Engineering Stanford University Stanford, CA United States; 7 Department of Education and Research Taipei City Hospital Taipei City Taiwan; 8 Center for Geriatrics and Gerontology Kaohsiung Veterans General Hospital Kaohsiung City Taiwan; 9 Department of Geriatric Medicine, School of Medicine National Yang Ming Chiao Tung University Taipei City Taiwan; 10 Division of Neurology, Department of Internal Medicine Kaohsiung Veterans General Hospital Kaohsiung City Taiwan; 11 Taipei Municipal Gan-Dau Hospital (Managed by Taipei Veterans General Hospital) Taipei City Taiwan

**Keywords:** digital mahjong, cognitive engagement intervention, gamification, cognitive function, healthy aging, magnetic resonance imaging

## Abstract

**Background:**

Gamified digital training may support long-term engagement in aging populations, yet randomized evidence linking behavioral effects to convergent neuroimaging outcomes remains limited.

**Objective:**

This study tested whether a home-based digital mahjong-based cognitive engagement intervention enhanced cognition in community-dwelling older adults and explored brain changes using multimodal magnetic resonance imaging (MRI).

**Methods:**

We conducted a single-blind, parallel-group randomized controlled trial in Taipei, Taiwan (July 2020-November 2021). Community-dwelling adults aged 55 years and older were randomized (1:1) to a 6-month digital mahjong-based intervention (two 30-minute sessions per week via an iPad app) with an incentivized walking component (Mi Band step tracking with in-game tokens) or no intervention. Primary MRI outcomes were gray matter volume, regional homogeneity (ReHo), fractional amplitude of low-frequency fluctuations (fALFF), and global brain connectivity. Secondary outcomes included cognition (Montreal Cognitive Assessment), physical activity (International Physical Activity Questionnaire), mental health (Brief Resilience Scale and Mandarin version of the Demoralization Scale), quality of life (EQ-5D with a visual analog scale), physical fitness and body composition, and blood biomarkers. Per-protocol analyses were performed. Voxel-wise imaging analyses used analysis of covariance in SPM12 with family-wise error (FWE) correction at the cluster level (*P*<.05). Sensitivity analyses controlling for MRI delay duration were conducted to assess robustness of findings.

**Results:**

In total, 60 participants were enrolled (n=30, 50% per group); 96.7% (29/30) in the intervention group and 90% (27/30) in the control group completed primary assessments. Compared with controls, the intervention group showed a greater improvement in Montreal Cognitive Assessment delayed recall (between-group difference=−0.79 points, 95% CI −1.538 to −0.042; *P*=.04). Neuroimaging revealed increased ReHo in the right insula (FWE-corrected *P*<.05) and decreased gray matter volume in the left frontal pole and orbitofrontal cortex, with reduced fALFF in the left frontal pole. Changes in delayed recall were positively associated with right insular ReHo and also showed significant associations with the right anterior cingulate gyrus (ReHo), left inferior frontal gyrus, and right paracingulate gyrus (fALFF; FWE-corrected *P*<.05 in all cases). In exploratory dose analyses, right insular ReHo correlated with total practice sessions (*r*=0.48; *P*=.009). Among physiological outcomes, the intervention group had a larger reduction in diastolic blood pressure (−3.50 mm Hg, 95% CI −6.911 to −0.091; *P*=.04), whereas the thyroid-stimulating hormone decreased but remained within the normal range. Sensitivity analyses confirmed that primary findings remained stable after controlling for MRI delay duration.

**Conclusions:**

This 6-month home-based digital mahjong intervention improved delayed recall and increased right insular ReHo. Integrating culturally familiar cognitive challenges with lifestyle engagement may be a feasible strategy associated with salience network–related functional changes and memory performance. Future large-scale trials should use active control conditions and factorial designs to rigorously isolate component-specific mechanisms.

**Trial Registration:**

ClinicalTrials.gov NCT05808426; https://clinicaltrials.gov/study/NCT05808426

## Introduction

### Background

Declines in physical and cognitive functions are major challenges for aging populations. Although multidomain interventions can help, maintaining long-term adherence remains difficult for many older adults. The World Health Organization healthy aging framework and the US National Academy of Medicine’s healthy longevity framework [1,2] indicate that effective strategies should target physical and cognitive health simultaneously rather than separately. Therefore, combining exercise with cognitive training is a recommended approach [3,4].

As neurodegenerative disorders become more prevalent, preventing functional decline is a priority [5,6]. However, purely physical or cognitive interventions often fail to maintain long-term engagement [7,8]. Previous studies have shown that multidomain interventions can promote healthy longevity; however, their benefits often diminish if participants do not continue the activities after the intervention ends [4,9]. Therefore, sustainable approaches to healthy aging must involve scientifically grounded, multifaceted strategies capable of maintaining participant engagement over the long term.

The interactive nature of digital gaming offers distinct advantages over traditional training, particularly regarding immediate feedback and user engagement. This interactivity makes digital gaming a promising tool for improving adherence in older adults [10] while also providing a feasible and relatively safe approach to support cognitive and physical activity [11]. There is recent evidence further suggesting that digital games may benefit cognitive performance and motor-related outcomes in older adults, particularly when the interventions are structured and appropriately tailored [12]. Research by Ding et al [13] and Zhang et al [14] has shown that digital versions of games such as mahjong can improve cognitive function, support mental health, and reduce cognitive decline in older adults. Mahjong, a traditional Chinese tile-based game, requires players to perform coordinated visual, mental, and cognitive tasks to achieve winning combinations. Studies indicate that both traditional and digital forms of mahjong can positively impact early-stage dementia in older adults [15].

Mechanistically, digital game training engages multiple cognitive domains, such as executive function, attention, and memory, through multitasking activities [16-18]. This process requires the brain to integrate functions across different regions to generate appropriate responses. Research has shown that, after multitasking video game training, older adults can achieve cognitive performance levels comparable to those of control participants aged 20 years [19]. Furthermore, complex game training not only improves cognitive performance in older adults but also induces significant changes in brain function and structure [20-23]. These neural changes demonstrate substantial neuroplasticity in response to targeted cognitive training, suggesting that mahjong-based game training could be an effective approach to promote brain plasticity [24]. Despite the importance of structural and functional brain markers in understanding cognitive aging, there have been few randomized controlled trials investigating the effects of digital mahjong gameplay on brain measures, cognitive function, and overall health.

We selected mahjong as the intervention for 3 reasons. First, it requires working memory, executive function, and attention, aligning with multidomain cognitive training principles [19,24]. Second, observational and experimental evidence links mahjong practice to preserved cognition [13-15]. Third, unlike laboratory tasks, mahjong is culturally familiar, which may enhance intrinsic motivation and adherence [10]. In contrast to traditional computerized cognitive training that isolates specific domains, our cognitive engagement strategy embeds training within a familiar context to improve ecological validity. We combined the core digital mahjong game with incentivized walking and gamified progression to sustain engagement. This design balances efficacy with real-world acceptability. While targeted serious games can effectively engage specific processes, our complementary approach prioritizes cultural relevance and sustainable engagement.

### Objectives

In this study, we aimed to evaluate whether a 6-month digital mahjong program could improve cognitive function and be associated with magnetic resonance imaging (MRI)–derived brain changes. We also tested whether adding a walking incentive would increase physical activity. Our study compared the intervention to a no-intervention control group across multiple domains, including MRI-derived brain measures, global and domain-relevant cognitive performance, overall health status, and blood biomarkers. We hypothesized that this integrated intervention would improve cognitive function and be associated with measurable changes in brain structure and function, reflecting the combined effects of sustained cognitive engagement and lifestyle modification.

## Methods

### Study Design

This was a single-blind, parallel-group randomized controlled trial registered at ClinicalTrials.gov (NCT05808426). Using a computer-generated random number sequence, we randomly assigned eligible participants in a 1:1 ratio to either an intervention group or a control group without any stratification. The control group received no intervention.

### Sample Size Estimation and Participants

We performed an a priori power analysis using G*Power (version 3.1.9.7) to determine the required sample size. The sample size was calculated based on Mini-Mental State Examination data reported in a previous study examining the effects of digital game interventions on cognitive function in older adults [20] in which the intervention and control groups showed mean scores of 28.87 (SD 1.64) and 27.72 (SD 1.60), respectively. We input these values into G*Power, and the resulting effect size was approximately 0.71. Assuming a 1-tailed *t* test with an α value of .05 and power (1 – β) of 0.8, the analysis indicated that 26 participants per group would be needed. To account for an anticipated attrition of approximately 10%, we targeted a recruitment of 30 participants in each group. Ultimately, 30 participants were enrolled in each group; by the end of the study, 96.7% (29/30) participants in the intervention group and 90% (27/30) in the control group completed the trial.

### Ethical Considerations

This study was approved by the institutional review board of National Yang Ming Chiao Tung University (YM108123F). All participants provided written informed consent in accordance with the Declaration of Helsinki. Participants were informed that they could withdraw from the study at any time, and all personal data were deidentified to ensure privacy and confidentiality. At the time of study design (2019-2020), it was genuinely unknown whether the intervention was efficacious. This study was approved as a minimal-risk proof-of-concept trial. A waitlist control design was not feasible due to resource constraints and COVID-19–related delays.

### Procedure

The program design, trial protocol, and intervention completion rate are provided in Multimedia Appendix 1 and Table S1 in Multimedia Appendix 2. Before starting the mahjong intervention, all participants in both groups completed baseline assessments, including questionnaires, physical examinations, blood tests, and brain MRI scans. The intervention content (the digital mahjong game and related procedures) was beta tested by the sponsoring company and piloted with a group of older adults not enrolled in the study.

This trial recruited community-dwelling adults aged 55 years and above in Taipei, Taiwan, between July 2020 and November 2021, corresponding to a 6-month intervention period for each participant. Data were collected at baseline and immediately after the intervention. Participants were enrolled via convenience sampling responding to advertisements posted in local health centers. This study was interrupted for approximately 3 months due to the COVID-19 pandemic. Participants assigned to the intervention group engaged in the digital mahjong program for the full 6-month period as planned. However, pandemic-related restrictions on physical activity caused both groups to experience a 6- to 8-month delay in undergoing postintervention brain MRI scans and other functional assessments.

Because the intervention was delivered individually and remotely, participants’ start and end dates varied. For example, the first participant underwent the baseline assessment on July 28, 2020; completed the 6-month digital mahjong game intervention by January 5, 2021; and then underwent the postintervention evaluation (including the MRI scan) on September 2, 2021 (approximately 8 months after intervention completion). The last participant finished all postintervention assessments by October 13, 2021. We paused the study for 3 months during Taiwan’s level 4 COVID-19 alert as restrictions prevented the delivery of Wi-Fi SIM cards. Make-up sessions were provided once restrictions eased. To mitigate potential decay of training effects due to the delay between intervention completion and postintervention MRI, we administered a 1-month “booster” retraining session to the intervention group immediately prior to the scheduled MRI assessment based on perceptual learning principles suggesting that brief refresher training can help consolidate and maintain cognitive gains.

### Randomization and Blinding

Participants were not informed of their group allocation, and they had no contact with the research team during the study. The research personnel were blinded to group assignments: all participant data were labeled with anonymous codes, so investigators could not discern which intervention a given participant received. Moreover, the staff members who delivered the intervention were separate from those who conducted outcome evaluations and data analysis. The research team also ensured that participants from different groups did not interact with one another throughout the trial.

### Intervention Program

The digital mahjong game was delivered via an iPad app (the Bonus Winner app), in which a single participant plays against 3 computer-controlled players, replicating the traditional game in a virtual format. The app was a commercially available platform adapted for research purposes with the developer’s cooperation. The key modification for this study was the implementation of a structured difficulty progression system based on response time constraints, allowing for parametric control over cognitive load. The research team provided an orientation to the digital mahjong platform and supplied each intervention group participant with an iPad. As part of the gamified design, participants could earn virtual bonus points through walking activity; therefore, each participant was given a Xiaomi Mi Band wristband to track their steps. A research assistant set up each participant’s device by installing a SIM card for Wi-Fi connectivity, instructed participants on using the mahjong app, and monitored their gameplay progress remotely. The assistant also demonstrated how the Mi Band’s recorded walking distance would be converted into in-game bonuses.

The digital mahjong game protocol (see Multimedia Appendix 1 for details) used a progressive difficulty system with real-time interactive gameplay. The game consisted of 4 levels, each with increasingly stringent response time limits:

- Level 1: 20-second response window (familiarization phase; month 1)
- Level 2: 7-second response window (standard training; months 2-6)
- Level 3: 5-second response window (advanced; optional)
- Level 4: 3-second response window (highest difficulty; optional)

To minimize ceiling effects and baseline heterogeneity, we excluded participants with regular mahjong experience (playing >3 times per year). However, we permitted basic rule familiarity to avoid the confounding load of learning game mechanics from scratch. All participants spent the first month at level 1 to ensure mastery of the digital interface before progressing. This graduated structure facilitates adaptation to increasing cognitive loads. To sustain motivation, we implemented a coin-based progression system that incentivized advancement while mitigating the frustration of losses. Comprehensive intervention details are provided in Multimedia Appendix 1.

### Outcomes

The primary outcome was defined as changes in brain structure and function as measured via MRI. MRI data acquisition and image processing for structural and functional analyses were conducted using standard protocols.

All MRI scans were performed on a 3T Siemens scanner at National Yang Ming Chiao Tung University. T1-weighted anatomical images were acquired using a 3D magnetization prepared rapid gradient echo sequence with the following parameters: repetition time=3500 ms; echo time=3.5 ms; inversion time=1100 ms; flip angle=7°; field of view=256 × 256 mm^2^; matrix size=256 × 256; voxel size=1.0 × 1.0 × 1.0 mm^3^; 192 sagittal slices; 1 excitation. Resting-state functional MRI (fMRI) data were collected using a T2*-weighted gradient-echo echo-planar imaging sequence using the following parameters: repetition time=2500 ms; echo time=30 ms; flip angle=90°; slice thickness=3.5 mm; image matrix size=64 × 64; field of view=222 × 222 mm; 43 interleaved slices (no gap); 200 volumes (acquisition of approximately 8 minutes and 27 seconds). All raw MRI scans were reviewed by an experienced researcher to ensure no anatomical abnormalities or substantial artifacts before further processing.

To characterize intervention-related changes in the brain, we evaluated four neuroimaging metrics: (1) gray matter volume (GMV; derived from structural MRI), which reflects regional GMV differences and was used as a structural MRI marker; (2) fractional amplitude of low-frequency fluctuations (fALFF; derived from resting-state fMRI), which reflects neural activity levels and their relationship to brain metabolic rate changes [25,26]; (3) regional homogeneity (ReHo; derived from resting-state fMRI), which measures the similarity of neural activity among neighboring brain regions [27]; and (4) global brain connectivity (GBC; derived from resting-state fMRI), which measures the interconnectivity of disparate brain regions, reflecting interactions among large-scale brain networks [28].

Detailed preprocessing methods for these MRI-derived features are provided in Multimedia Appendix 1. Whole-brain voxel-wise maps for GMV, ReHo, fALFF, and GBC were generated for statistical analysis.

Secondary outcomes were assessed using validated tools across cognitive, physical, mental, and quality of life domains. Cognitive function was measured using the Montreal Cognitive Assessment (MoCA), a 30-point cognitive screening tool covering multiple domains (orientation, memory, executive and visuospatial function, language, abstraction, naming, attention, and clock drawing) [29,30]. The MoCA takes approximately 10 to 12 minutes to administer, and scores of 26 or higher are considered normal. It has strong psychometric properties and is widely used as a reliable screening instrument for dementia.

Physical activity was measured using the short form of the International Physical Activity Questionnaire (IPAQ; 7-day recall) [31,32]. This questionnaire captures physical activity across work, household, transportation, and leisure domains at 3 intensity levels. Weekly physical activity was calculated in metabolic equivalent of task (MET) minutes by summing the time spent walking (3.3 METs), in moderate-intensity activities (4.0 METs), and in vigorous-intensity activities (8.0 METs). The short form of the IPAQ has excellent content validity (content validity index=0.994) and adequate test-retest reliability (r=0.67).

Mental health was evaluated using 2 self-report scales: the Brief Resilience Scale [33] and the Demoralization Scale (Mandarin version) [34-36]. The Brief Resilience Scale is a 6-item questionnaire measuring an individual’s resilience, with items rated on a 5-point Likert scale (1=“strongly disagree”; 5=“strongly agree”). After reverse scoring the 3 negatively worded items, responses are averaged, with higher scores indicating greater resilience. The Mandarin version of the Demoralization Scale consists of 24 items rated from 0 (“never”) to 4 (“all of the time”), with a total score of 30 or higher indicating demoralization syndrome (higher scores reflect greater severity of demoralization).

Quality of life was assessed using the EQ-5D questionnaire [37,38]. This instrument evaluates 5 domains: mobility, self-care, usual activities, pain and discomfort, and anxiety and depression, each on a 3-level scale (“no problems,” “some or moderate problems,” and “extreme problems”). Participants also rated their overall health on the EQ-5D’s visual analog scale from 0 (worst imaginable health) to 100 (best imaginable health). The EQ-5D (including the visual analog scale) is a widely used and validated measure of general health status in older adult populations.

Additionally, we administered standard physical fitness tests, measured body composition using an InBody S10 bioimpedance analyzer, and collected blood samples for a panel of biochemical markers. Other recorded information included participants’ demographic characteristics, health-related behaviors (eg, tobacco and alcohol use), and any self-reported physician-diagnosed medical conditions.

### Statistical Analysis

All analyses were conducted on a per-protocol basis comparing the intervention and control groups’ outcomes from baseline to the postintervention time point. Continuous variables were summarized as means and SDs and compared between groups using appropriate significance tests (2-tailed Student *t* test for parametric data or Mann-Whitney *U* test for nonparametric data). Categorical variables were summarized as counts and percentages and compared using chi-square or Fisher exact tests. Statistical significance was set at a 2-sided *P* value of less than .05 (95% CIs that excluded the null value). Data were analyzed using SPSS Statistics (version 25.0; IBM Corp).

For primary outcomes derived from neuroimaging measures, we used a difference-in-differences approach to compare changes from baseline to the 6-month follow-up between groups. Specifically, after preprocessing, individual posttest-minus-pretest change maps were estimated for each MRI metric by subtracting each participant’s baseline image map from the corresponding postintervention image map. These voxel-wise change maps were then entered into group-level analyses in SPM12 to test whether longitudinal changes differed between the intervention and control groups. As the dependent variables were posttest-minus-pretest change maps, baseline MRI maps were incorporated into the calculation of within-participant change rather than entered as separate covariates. We used analysis of covariance models to test for intervention effects on MRI outcomes adjusting for relevant covariates (baseline age, sex, and intracranial volume for structural measures and age and sex for functional measures). To correct for multiple voxel-wise comparisons, we applied a cluster-level family-wise error (FWE) correction at a *P* value of less than .05 using the AFNI program’s 3dClustSim (version 20.3.03) with an initial voxel-wise threshold of a *P* value below .005. The minimum cluster size for significance was determined separately for each imaging metric (eg, 166 voxels for GMV, 57 voxels for ReHo, 19 voxels for fALFF, and 10 voxels for GBC).

To assess the robustness of our neuroimaging findings to variable delays between intervention completion and postintervention MRI assessment, we conducted sensitivity analyses including MRI delay duration as an additional covariate in all analysis of covariance models. This approach allowed us to evaluate whether observed brain changes were confounded by the timing of postintervention assessments. Details and results of these sensitivity analyses are provided in Multimedia Appendix 3.

To explore dose-response relationships in the intervention group, we examined whether the amount and type of mahjong practice correlated with brain changes. We first extracted the mean values from each participant’s significant brain clusters (identified in the GMV, ReHo, fALFF, and GBC analyses). Then, using partial Pearson correlations (controlling for age, sex, and MRI delay duration in sensitivity analyses), we tested associations between these brain change metrics and the number of completed mahjong sessions at various difficulty levels (total sessions and sessions with 20-, 7-, 5-, and 3-second response time limits). The significance for these correlations was evaluated at a false discovery rate–corrected level of a *P* value below .05.

We also investigated whether improvements in cognitive scores were associated with neuroimaging changes. For each participant, we computed the change (baseline to posttest) in cognitive performance (eg, MoCA delayed recall) and extracted the corresponding change maps for brain outcomes. Using SPM12, we built multiple linear regression models to test the association between cognitive improvement and brain changes adjusting for age and sex (and also adjusting for baseline intracranial volume for structural MRI measures). These regression analyses did not yield any significant voxel-wise associations at the predefined threshold (cluster-level FWE *P*<.05). Further details of all statistical procedures are provided in Multimedia Appendix 1.

## Results

### Participants

**Figure 1:**
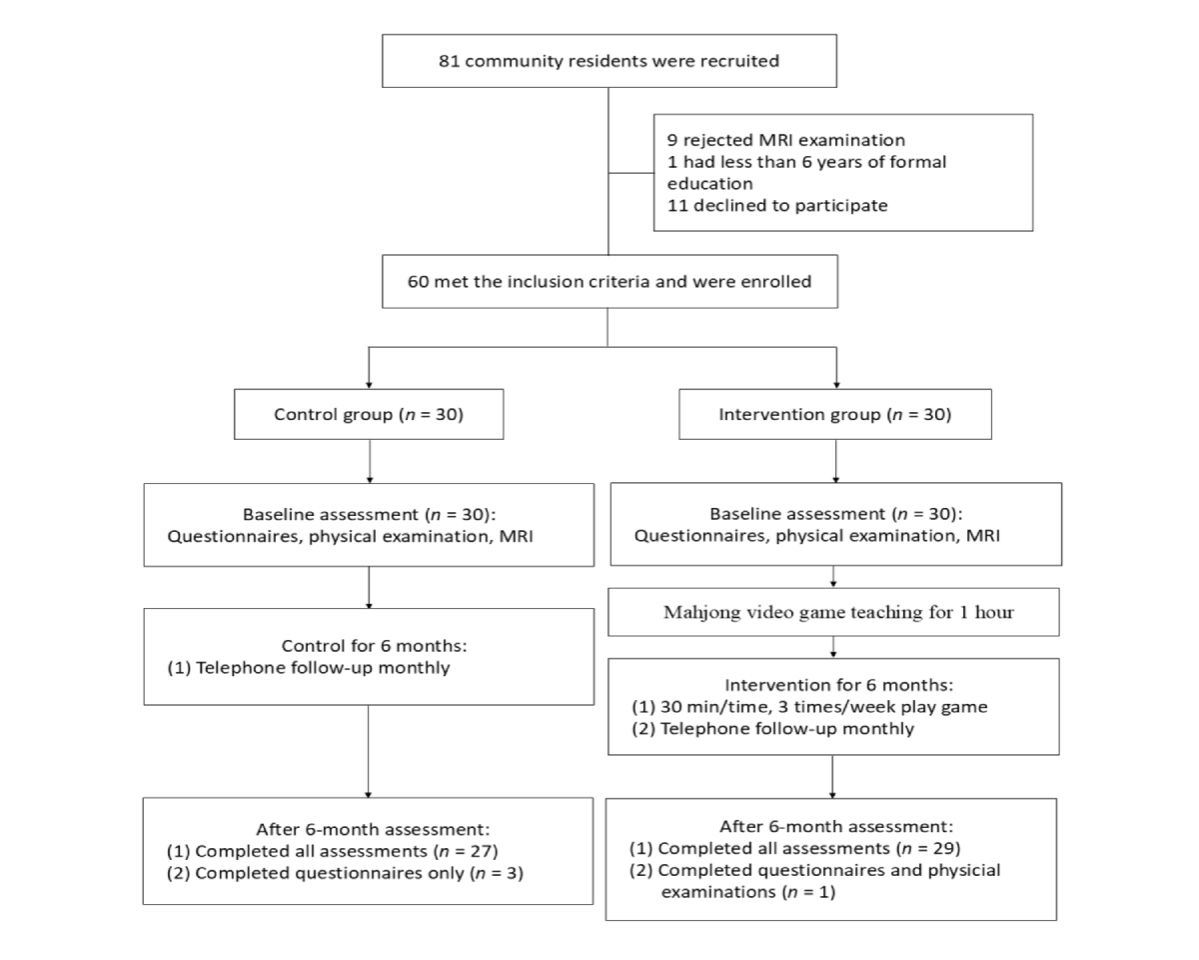
CONSORT (Consolidated Standards of Reporting Trials) flow diagram indicating the number of participants enrolled, the randomization process, and the study flow. MRI: magnetic resonance imaging.

Figure 1 shows the participant flow from initial screening and enrollment to random allocation (30 participants per group). Participant characteristics included a mean age of 64.45 (SD 5.23) years in the intervention group and 68.07 (SD 5.78) years in the control group (*P*=.27). The intervention group consisted of 34.5% (10/29) male participants, whereas the control group comprised 40.7% (11/27) male participants (*P*=.78). The groups did not differ significantly on baseline physical activity (IPAQ), physical fitness, or body composition measures, supporting comparability prior to randomization. Additional baseline demographic characteristics for each group are provided in Table S2 in Multimedia Appendix 2.

### Intervention Engagement

Detailed engagement metrics are provided in Table S1 in Multimedia Appendix 2. Briefly, participants in the intervention group completed a mean total of 825.2 (SD 615.0; range 289-3055) sessions over the 6-month period. All participants in intervention group (30/30, 100%) completed the familiarization phase (level 1; 20-second response window), 100% (30/30) progressed to level 2 (7-second response window), 100% (30/30) advanced to level 3 (5-second response window), and 86.7% (26/30) attempted the highest difficulty (level 4; 3-second response window). The mean number of daily steps recorded via Mi Band was 8826 (SD 3205.4; range 85.0-19,543.5). Protocol adherence (completing ≥80% of scheduled sessions) was achieved by all participants, with a mean completion rate of 94.44% (SD 6.09%; range 83.33%-100%).

### Primary Outcome

**Figure 2:**
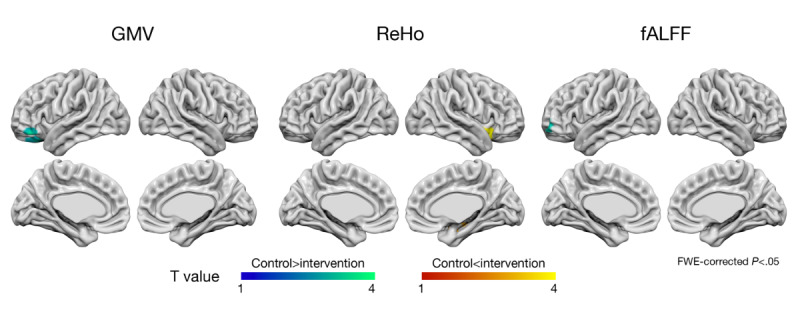
Brain measure changes between the control and intervention groups. Hot (red-yellow) regions reflect a significantly increased change in the intervention group. Cold (blue-green) regions reflect a significantly decreased change in the intervention group. fALFF: fractional amplitude of low-frequency fluctuations; FWE: family-wise error; GMV: gray matter volume; ReHo: regional homogeneity.

At the 6-month follow-up, the intervention group exhibited significant intervention-related changes in brain structure and function compared to controls. In voxel-wise analyses (Figure 2; Table S3 in Multimedia Appendix 2), the intervention group showed reduced GMV in the left frontal pole and left orbitofrontal cortex, as well as decreased fALFF in the left frontal pole. Conversely, ReHo in the right insula was significantly increased in the intervention group (cluster-level FWE *P*<.05). Notably, none of these brain changes were significantly associated with the frequency of digital mahjong gameplay during the intervention.

**Figure 3:**
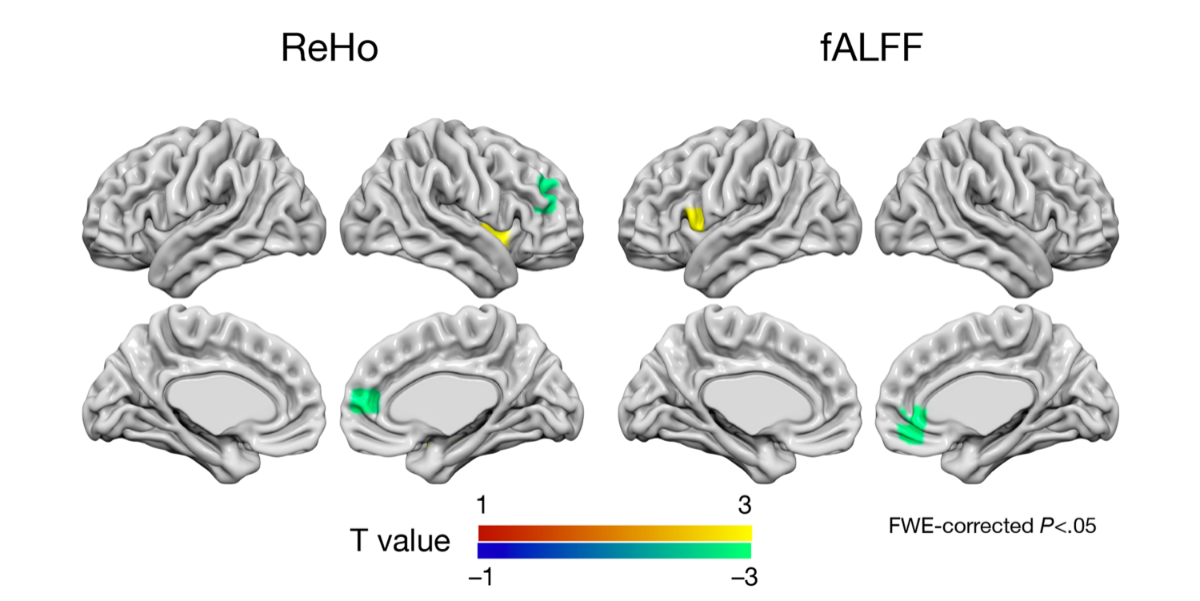
Associations between changes in intrinsic functional brain measures and improvements in delayed recall performance. Multiple linear regression analyses were performed to examine the associations between changes in intrinsic functional brain measures (regional homogeneity [ReHo] and fractional amplitude of low-frequency fluctuations [fALFF]) and improvements in Montreal Cognitive Assessment (MoCA) delayed recall scores. Analyses were adjusted for age and sex. Hot (red-yellow) regions reflect a significantly positive correlation with the cognition outcome. Cold (blue-green) regions reflect a significantly negative correlation with the cognition outcome. FWE: family-wise error.

**Figure 4:**
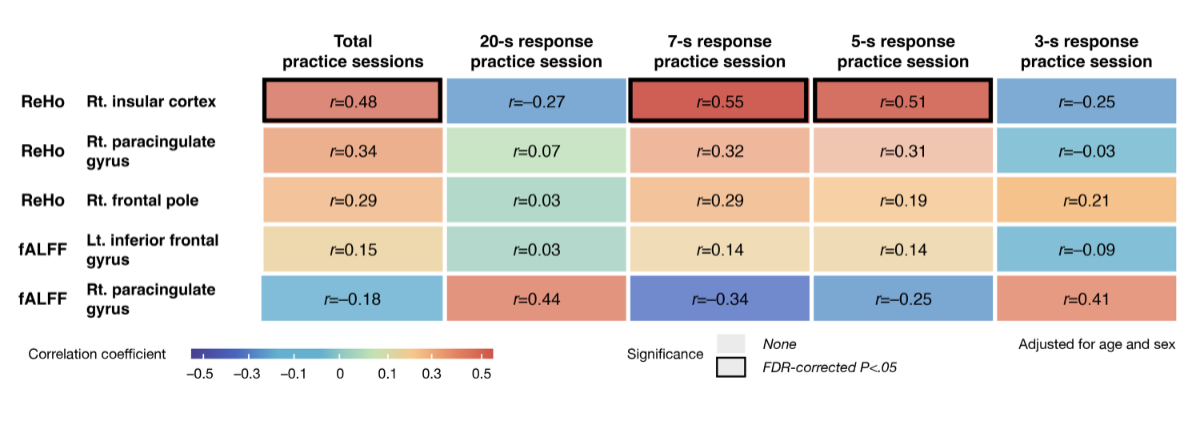
Associations between brain signature changes and the number of mahjong training sessions completed at different response-time levels. Relationships were estimated using partial Pearson correlation analysis accounting for age and sex. Significant correlations are marked with a black box (false discovery rate [FDR]–corrected *P*<.05). fALFF: fractional amplitude of low-frequency fluctuations; Lt: left; ReHo: regional homogeneity; Rt: right.

The regression analyses examining cognitive-brain relationships indicated that changes in GMV were not significantly related to improvements in cognitive performance (eg, MoCA delayed recall). In contrast, changes in MoCA delayed recall scores were significantly associated with functional connectivity changes: a greater improvement in delayed recall was associated with an increase in ReHo in the right insula and a decrease in ReHo in the right paracingulate gyrus and right frontal pole (cluster-level FWE *P*<.05 in all cases; Figure 3; Table S4 in Multimedia Appendix 2). Similarly, for fALFF, higher delayed recall gains were linked to increased fALFF in the left inferior frontal gyrus and decreased fALFF in the right paracingulate gyrus (FWE *P*<.05). Finally, intervention participants who completed more training sessions, especially those with faster response times (7- and 5-second limits), exhibited higher ReHo in the right insula. In exploratory dose analyses, right insular ReHo correlated with total practice sessions (*r*=0.48; *P*=.009; Figure 4).

Sensitivity analyses controlling for MRI delay duration confirmed the robustness of the primary findings. Detailed sensitivity analysis results are provided in Multimedia Appendix 3. Group-level results for GMV and fALFF remained consistent with those of the original analyses (Table S1 and Figure S1 in Multimedia Appendix 3). Although the ReHo activation pattern in the right insula did not survive strict cluster-level FWE correction in the adjusted model due to reduced statistical power, the effect remained visible at a relaxed threshold (uncorrected *P*<.05; cluster of >100 voxels). Importantly, the brain-behavior correlations and dose-response relationships remained significant after controlling for the delay, with peak coordinates identical to those of the original analysis (Table S2 and Figures S2 and S3 in Multimedia Appendix 3).

### Secondary Outcomes

Table S5 in Multimedia Appendix 2 summarizes the between-group comparisons for cognitive function, physical activity, psychological measures, quality of life, body composition, and blood biomarkers. The intervention group demonstrated a significantly greater improvement in MoCA delayed recall from baseline than the control group (between-group difference=−0.79, 95% CI −1.538 to −0.042; *P*=.04). Both groups completed identical MoCA assessments at baseline and follow-up under the same conditions. Importantly, the improvement was specific to delayed recall rather than domains more susceptible to practice effects (eg, orientation and naming), suggesting domain-relevant gains aligned with the memory demands of the intervention rather than generalized practice effects. The intervention group also reported a reduction in weekday sitting time relative to baseline, but this decrease was not significantly different from that in the control group (+71.7 minutes, 95% CI −7.8 to 141.1; *P*=.08). There were no significant between-group differences in resilience, demoralization, or quality of life outcomes.

Physical examination results showed that the intervention group had a significant reduction in diastolic blood pressure (3.50 mm Hg, 95% CI, 0.091-6.911; *P*=.04). Although the control group showed greater decreases in muscle mass measurements (right leg, left leg, total limb muscle mass, and relative appendicular skeletal muscle), these differences were not statistically significant between groups (Table S5 in Multimedia Appendix 2).

Biochemically, the intervention group showed a significant decrease in serum thyrotropin levels compared to the control group (−0.545 µIU/mL, 95% CI −1.018 to −0.072; *P*=.02). As all participants’ thyrotropin level values remained within the normal range, the clinical significance of this drop is unclear. Total cholesterol levels changed slightly in the intervention group relative to controls, but there were no corresponding significant changes in high-density lipoprotein or low-density lipoprotein cholesterol.

Finally, exploratory hierarchical regression analyses (Table S6 in Multimedia Appendix 2) were conducted to investigate how the extent of engagement with the intervention related to the secondary outcomes. These analyses suggested that participants with longer average daily play time tended to have greater reductions in total cholesterol (*P*=.03) and those who frequently played with the 20-second response window tended to have higher high-density lipoprotein cholesterol (*P*=.047). Additionally, the total number of incentivized walking steps was positively correlated with thyroid-stimulating hormone levels at 7 months (*P*=.03).

## Discussion

### Principal Findings

Our study showed that a home-based digital mahjong-based cognitive engagement intervention significantly improved delayed recall in older adults. We also observed functional changes in the right insula, suggesting that the training involved specific brain networks. While many health interventions are effective in the short term, adherence often declines over time. Our study demonstrates that a culturally adapted digital tool can sustain engagement and improve delayed recall. Gamification has emerged as a promising approach to encourage healthy behaviors, but its long-term efficacy relative to more traditional interventions remains to be fully elucidated.

The findings from our trial indicate that the digital mahjong-based cognitive engagement intervention significantly enhanced delayed recall ability and showed accompanying changes in brain functional measures. The observed effects reflect the combined intervention package (digital mahjong, incentivized walking, and gamification) rather than any single component. In particular, the most challenging training sessions (with 7- and 5-second response time limits) showed the strongest associations with right insular ReHo. These findings align with those of previous research showing that cognitive training can enhance memory [39,40]. Our study adds exploratory neuroimaging findings to behavioral findings. Specifically, we observed increased ReHo in the right insula. The preferential engagement of this region during high-intensity (5- and 7-second) sessions may reflect increased demands on decision urgency and error monitoring. This pattern is consistent with the potential involvement of the salience network in mediating rapid switching between internal processing and external stimuli. However, because the right insular ReHo finding was attenuated in sensitivity analyses after controlling for MRI delay duration, this result should be interpreted with caution given its sensitivity to model specification.

We also noted significant GMV reduction in the frontal pole, a region critical for working memory [41,42]. Given the concurrent improvement in delayed recall performance, this decrease may be compatible with experience-related structural reorganization or enhanced neural efficiency rather than necessarily indicating maladaptive atrophy. This finding is consistent with those of recent randomized controlled trials, which similarly found no evidence of macroscopic structural growth (volume expansion) despite rigorous cognitive training. Together, these findings support the hypothesis that functional connectivity alterations (such as ReHo) may precede or accompany structural changes [43]. Behaviorally, we further noticed that participants relied heavily on in-game cues during the most challenging (7 and 5 seconds) response time sessions. This suggests that the high cognitive load necessitated external support, reinforcing the possibility that intensive cognitive engagement may be associated with changes in specific brain networks.

This trial demonstrated that a gamified cognitive engagement intervention can significantly improve cognitive performance (specifically delayed recall) in older adults, corroborating previous findings that cognitively stimulating games such as mahjong yield cognitive benefits [13,14]. Our study uniquely combined the digital game with an incentive program: participants earned virtual tokens for in-game rewards by walking, thereby motivating both cognitive engagement and physical activity. Unfortunately, the onset of the COVID-19 pandemic likely blunted the full benefits of this design. As infection concerns grew, participants became less willing to go outside, reducing their walking activity despite the token incentive. Indeed, pandemic-related restrictions and safety measures have been shown to adversely affect older adults’ physical, cognitive, and mental well-being [44,45]. Although Taiwan experienced a relatively mild outbreak, these disruptions probably limited the effectiveness of our walking-based token system to simultaneously promote exercise and cognitive training.

Mahjong gameplay has also been linked to mental health benefits (eg, lower depression and better well-being), presumably through increased social interaction [46]. In our study, however, the digital format did not yield measurable gains in physical performance, psychological resilience, demoralization, or quality of life relative to controls. One explanation for the absence of these effects is a possible ceiling effect: our participants were relatively healthy at baseline, leaving little room for improvement on those measures. Moreover, both groups experienced comparable pandemic-related stress during the trial, which could have obscured subtle intervention benefits. Despite these null findings, our intervention did produce robust gains in delayed recall along with corresponding brain changes. The integrated walking incentive successfully promoted physical activity, with participants averaging 8826 (SD 3205.4) steps per day despite pandemic restrictions. This sustained activity level suggests that the gamified token economy remained effective in motivating physical movement even under constrained environmental conditions, which likely contributed to improvements in cardiovascular indicators. In fact, the intervention group showed significant reductions in diastolic blood pressure and total cholesterol, outcomes consistent with increased daily activity levels [47,48].

Unlike targeted serious games that isolate specific cognitive processes, our study prioritized ecological validity and cultural acceptability. Mahjong engages multiple domains simultaneously, mirroring real-world cognitive demands. We acknowledge the trade-off between the mechanistic precision of laboratory tasks and the implementability of lifestyle interventions. However, culturally meaningful activities such as digital mahjong often promote better long-term adherence than abstract computerized cognitive training programs. This sustained engagement may yield greater cumulative benefits over time even if the training target is less precise. Future studies should directly compare serious games with culturally adapted activities to guide recommendations for diverse populations.

### Limitations

Our study has several limitations. First, the lack of an active control group limits our ability to exclude placebo effects. Participants in the intervention group received staff attention, technology, and incentives, all of which could trigger Hawthorne or expectancy effects. However, the observed gains were most evident in delayed recall, a core demand of mahjong, rather than generalized improvements. This pattern, combined with the neuroimaging correlations, suggests but does not establish intervention-specific effects. Future studies should use active controls, such as nonstrategic games or educational activities [43], to isolate the specific effects of mahjong training.

Second, COVID-19 restrictions necessitated a 6- to 8-month delay between intervention completion and MRI assessment. To mitigate potential signal decay, we administered a 1-month booster session prior to scanning and controlled for delay duration in sensitivity analyses [43]. Although these analyses confirmed the stability of brain-behavior correlations, we acknowledge that the observed neuroimaging differences may partially reflect consolidation processes or environmental factors during the hiatus rather than acute training effects alone.

Third, our intervention combined digital mahjong with incentivized walking, preventing us from isolating the specific contribution of each component. Consequently, the reported effects reflect the aggregate intervention. However, there are 2 lines of evidence suggesting that cognitive training was the primary driver of the observed neuroimaging differences. First, we found a specific dose-response relationship between mahjong practice intensity and right insular ReHo, supporting a link between cognitive training intensity and neural changes. Second, behavioral improvements were specific to delayed recall rather than physical function measures. While these findings support the specificity of the cognitive training, we cannot rule out synergistic effects between the cognitive and physical elements.

Fourth, because the incentive (token) program was applied only to the intervention group, we could not evaluate whether control participants might also have increased their physical activity without incentives. That said, it is generally recognized that specific incentives are often required to induce meaningful behavior change in health promotion programs.

Fifth, the COVID-19 pandemic and resulting restrictions led to a temporary suspension of the trial, interrupting the link between walking and token rewards. Nevertheless, the core element of our intervention, the digital mahjong game, remained relatively resilient to pandemic disruptions. Notably, previous neuroplasticity trials have found that training gains can persist for 3 to 6 months after an intervention even if performance at follow-up is somewhat below the posttraining peak [19,49]. To help sustain the cognitive benefits in our study, we administered a 1-month “booster” retraining to the intervention group approximately 1 month before the final assessment following perceptual learning principles to reinforce memory traces [50]. Despite these efforts, the pandemic-related delays postponed our postintervention evaluations, which may have attenuated the observed effects. Therefore, our results could be underestimating the true efficacy of the intervention.

We were also concerned about this study not monitoring participants’ use of the mahjong app after completion of the intervention; therefore, the sustainability of engagement, continued gameplay habits, and potential risks of excessive use could not be evaluated. Future studies should include longitudinal follow-up assessments to examine both the maintenance of intervention benefits and the promotion of appropriate, balanced use of gamified digital health apps.

### Conclusions

This randomized controlled trial suggests that a digital mahjong-based cognitive engagement intervention improved delayed recall and was associated with MRI-derived functional changes (right insular ReHo) in middle-aged and older adults. We also observed secondary benefits for diastolic blood pressure and total cholesterol. These findings support the viability of integrating culturally meaningful activities into lifestyle interventions to promote healthy aging. Future large-scale trials should use active control conditions and factorial designs. Such rigor is necessary to distinguish the specific effects of cognitive training from those of physical activity and optimize these tools for routine geriatric care.

## Appendix

Multimedia Appendix 1 Trial protocol and statistical analysis plan.

Multimedia Appendix 2 Supplementary tables describing the digital mahjong intervention protocol, participant characteristics, magnetic resonance imaging findings, intervention outcomes, and exploratory analyses.

Multimedia Appendix 3 Sensitivity analysis.

Multimedia Appendix 4 CONSORT checklist.

## Abbreviations

CONSORT: Consolidated Standards of Reporting Trials
fALFF: fractional amplitude of low-frequency fluctuations
fMRI: functional magnetic resonance imaging
FWE: family-wise error
GBC: global brain connectivity
GMV: gray matter volume
IPAQ: International Physical Activity Questionnaire
MET: metabolic equivalent of task
MoCA: Montreal Cognitive Assessment
MRI: magnetic resonance imaging
ReHo: regional homogeneity

## Data Availability

The data that support the findings of this study are available from the corresponding author on reasonable request.
