# Supplementary material for: Digital Mahjong-Based Cognitive Engagement Intervention for Older Adults: Randomized Controlled Trial
Source: JMIR Aging. 2026 Jul 23;9:e85339. doi: 10.2196/85339 (PMC13448193; doi:10.2196/85339)
Supplement: Supplementary file 2 [file aging_v9i1e85339_app1.docx]

**Multimedia Appendix 1. Trial protocol and statistical analysis plan.**

**1) Trial Overview**

- **Design & Registration:** Single-blind, parallel-group randomized controlled trial; registered at ClinicalTrials.gov (NCT05808426).
- **Setting & Timeline:** Taipei, Taiwan; recruitment and intervention occurred from July 2020 to November 2021 with COVID-related interruptions (see Section 7).
- **Ethics:** Approved by the institutional review board; written informed consent obtained.
- **Participants:** Community-dwelling adults aged **≥55** years.
- **Randomization & Allocation:** 1:1 allocation to Digital Mahjong (DMG) intervention vs no-intervention control; computer-generated block randomization; allocation concealed from assessors (single-blind).
- **Analysis Set:** **Per-protocol** analyses for primary outcomes, consistent with the main text.

**2) Eligibility & Recruitment**

**Inclusion criteria**

1. Age ≥ 55 years old.

2. More than 6 years of formal education.

3. Can communicate in Mandarin and Taiwanese.

4. Can accept MRI examination without claustrophobia.

5. Can understand the research process, meet the requirements of the designed intervention, and can sign the consent form.

**Exclusion criteria:**

1.	Unable to communicate adequately with study staff.

2.	Malignancy undergoing active chemotherapy.

3.	Life expectancy <12 months.

4. Engaged in Mahjong games more than three times per year in the past three years.

**Recruitment:** Advertising in community centers.

**3) Intervention Details (reproducibility essentials)**

- **Platform & Opponents:** DMG delivered via an iPad app; participants played against three computer-controlled opponents (no human multiplayer).
- **Dose & Schedule:** Two 30-minute sessions per week for 6 months (scheduled; monitored via in-app logs).
- **Difficulty Tiers & Progression:** Participants begin at the easiest level, which provides a 20-second window for each move. They then advance to more challenging levels that require moves within 7 seconds, 5 seconds, and finally 3 seconds at the highest difficulty. This graduated response-time structure enables players to acclimate to rising cognitive demands as the game progresses
- **Incentivized Walking Component:** Daily steps tracked by Mi Band; in-game tokens awarded according to step ranges. Tokens unlock in-app cosmetic rewards and game content; no monetary value.
- **Intervention Protocol:**
  - Digital Mahjong, through an iPad with a bonus winner APP, refers to sing player matching tiles with the other three computer-matching players instead of a physical tabletop game. The research team provides an initial section to introduce digital Mahjong and provide an iPad for each participant in the experimental group. We designed to use walking distance to trad bonus, thus, mi band wristband to send the record to the platform was provided for each participant in the study. The research assistant also installed Sim Card with Wifi and demonstrated how to use APP by iPad and tracked the status of Mahjong game. In addition, the research assistant showed that the function of mi band and testing walking distance to trad bonus.
  - Traditionally, Mahjong is a competing game by 4 people, each one in front of one side of a square table physically. Mahjong uses various combinations to form a specific kind, then, integrated various kinds into a set. There are 136 tiles classically and add additional 8 tiles (four flower tiles and four-season tiles), as a sum of 144 tiles. For 136 tiles, including 36 characters, 36 bamboo tiles,36 circles (suits),16 wind tiles, and 12 dragons. The player gets all 14 tiles and they need to categorize them into four sets and one pair. Sets of tiles are called pung or chow, pung refers to three identical mahjong tiles and chow refers to three consecutive numbers. For identical tile pairs which can be any. The game requires you to make identical sets and matching pairs from these tiles to complete the set. To win the game, memory skill and logical thinking are essential.
  - The DMG consists of 4 levels of responding time from easy to difficult with live interaction, which are responds within 20 second (level one), 7 second (Level 2), 5 second (level 3) and 3 second (level 4). The 10000 coins for the participants in experimental group are provided. The participants at least play three times a week with a duration of more than 24 mins for 6 months. At the first month of intervention, all the participants play Mahjong at level one to familiar with the game rule. After first month, the participants play Mahjong at least start from level 2 within 7 second responds time. There are entry criteria in different level to encourage player move to higher level, including player hold less than 3000 coins can enter level one and two. At the same time, player had more than 1000 coin can check in 5 responds time practice sessions and 3000 coins for 3 responds time practice sessions. Even the player lost the games would not own coins, as such, encouraging the participants can play with joy. Also, each player can receive more coins per winning round at higher level of Mahjong games to motivate the participants improve their performance. The game rule is 50 coins for 7 responds time practice sessions, 100 coins for 5 responds time practice sessions and 300 coins for 3 responds time practice sessions when the player win. Besides coins from per winning, extra coins would be given while the payer win according to specific tilter. The player can win extra 20 coins with specific tiler in level 1 and 2, win 30 coins in level 3 and 100 coins in level 4.
  - To promote physical activity by walking steps, the program design provides a bonus by walking steps, estimated by wearable devices (mi smart band-4) from the second month to the end of the program. If the participant walked with 5000~9999 steps per day win 800 bonus, 10000~14999 steps per day win 1200 bonus,15000~19999 steps per day win 1600,20000 steps per day win 2000 bonus, The completion rate was accounted by the expected level of difficulty and participation rate. The completion rate was varied, ranging from 50% to 100%, might be due to long distant digital Mahjong with no in person interaction.

**4) MRI Acquisition and Image Processing Details**

- **MRI Scanner:** All scans were acquired on 3T Siemens systems at National Yang-Ming Chiao-Tung University. A system upgrade from Siemens Tim Trio to Prisma Fit occurred between January and September 2021, with baseline scans on Trio and follow-up scans on Prisma Fit. The main static magnet was maintained to minimize system effects.
- **T1-weighted Structural Imaging:** 3D-MPRAGE sequence with TR/TE/TI = 3500/3.5/1100 ms, flip angle = 7°, voxel size = 1.0 × 1.0 × 1.0 mm³, 192 sagittal slices without gaps.
- **Resting-state fMRI:** Gradient-echo EPI with TR/TE = 2500/30 ms, flip angle = 90°, slice thickness = 3.5 mm, matrix = 64 × 64, 43 interleaved slices, 200 volumes (8 min 27 s acquisition time).
- **Image Preprocessing:**
  - **Structural Data (Gray Matter Volume):** Preprocessing used SPM12 with DARTEL-VBM procedure: bias correction → tissue segmentation → normalization to MNI space → modulation → smoothing (6-mm FWHM). Total intracranial volume was calculated as a covariate.
  - **Functional Data (ReHo, fALFF, GBC):** Preprocessing pipeline (AFNI, FSL, SPM12) included: removal of first 10 volumes → motion correction → slice-timing correction → brain extraction → despiking/detrending → nuisance regression (Friston 24-motion parameters, WM/CSF signals) → normalization to MNI (2-mm voxels).
- **Derived Functional Indices:**
  - **ReHo:** Kendall's coefficient of concordance across 26 nearest neighbors after bandpass filtering (0.01-0.08 Hz); z-standardized and smoothed (6-mm FWHM).
  - **fALFF:** Ratio of low-frequency (0.01-0.08 Hz) to full-band power (0-0.2 Hz); z-standardized after smoothing (6-mm FWHM).
  - **GBC:** Voxel-to-whole-brain correlation after filtering and smoothing (6-mm FWHM); resampled to 3-mm for computation efficiency; Fisher z-transformed.
- **Quality Control:** Exclusion or censoring thresholds applied for excessive motion and artifacts; visual QC conducted for segmentation/normalization.

**5) Deviations & Mitigation (COVID-19 context)**

- **Disruptions:** Government restrictions led to temporary pauses in on-site visits and delayed post-intervention MRI/assessments. Outdoor walking declined despite incentives.
- **Mitigation:** Rescheduling with identical protocols; remote check-ins for safety and engagement; a one-month “booster” DMG refresher administered pragmatically prior to final assessments (not pre-registered).
- **Interpretation:** Timing shifts may have attenuated effect sizes; estimates are likely conservative.

**6) Measurement Tools of secondary outcomes**

| **Construct** | **Instrument** | **Items/Time** | **Scoring (brief)** | **Reference(s)** |
| --- | --- | --- | --- | --- |
| Global cognition | MoCA | ~10–12 min | Total 30; higher=better | (main text refs) |
| Physical activity | IPAQ (short) | 7-day recall | MET-min/week by intensity |  |
| Resilience | BRS | 6 items | Mean of 6; higher=better |  |
| Demoralization | DS-MV | 24 items | Total score; ≥30 indicative |  |
| QoL | EQ-5D + VAS | <5 min | 5 domains (3 levels) + 0–100 VAS |  |
| Fitness/Body composition | Standard tests; InBody S10 | — | Standard indices |  |
| Biomarkers | Blood panel | — | Standard assays |  |

Full scoring manuals are available from instrument developers; the main text provides targeted details and citations.
