# Supplementary material for: Digital Mahjong-Based Cognitive Engagement Intervention for Older Adults: Randomized Controlled Trial
Source: JMIR Aging. 2026 Jul 23;9:e85339. doi: 10.2196/85339 (PMC13448193; doi:10.2196/85339)
Supplement: Supplementary file 3 [file aging_v9i1e85339_app2.docx]

**Multimedia Appendix 2. Supplementary tables.**

Table S1. Description and completion rates of digital Mahjong game

Table S2. Demographic Information for participants in each group

Table S3. Anatomical regions of structural and functional change between group

Table S4. Mahjong performance and secondary outcomes

Table S5: Changes in MRI, cognitive performance, physical assessment, and bioassay findings

Table S6. Anatomical regions of intrinsic functional measures with delayed recall score

Circumference

This supplementary material has been provided by the authors to give readers additional information about their work.

**Table S1: Description and completion rates of digital Mahjong game**

|  | Description of session | Participants who completed session (n=30) |
| --- | --- | --- |
| Pre- intervention  Baseline data collection | Baseline data collection including questionnaire, physical examination, lab works and MRI | 30 (100%) |
| Intervention at first month | Three times a week with no specific level for 24 minutes each time | 27(90%) |
| Intervention at second month | Three times a week for 24 minutes each time, and play level two at least 4 rounds | 23 (77%) |
| Intervention at third month | Three times a week for 24 minutes each time, and play level two at least 8 rounds | 23 (77%) |
| Intervention at forth month | Three times a week for 24 minutes each time, and play level two at least 12 rounds | 24 (80%) |
| Intervention at fifth month | Three times a week for 24 minutes each time, and play level two at least 16 rounds | 26 (87%) |
| Intervention at sixth month | Three times a week for 24 minutes each time, and play level two at least 20 rounds | 27 (90%) |
| Post-intervention | Post-intervention data collection including questionnaire, physical examination, lab works and MRI | 29 (97%) |

Level 1: less 20 second responds time,

Level 2: less 7 second responds time;

Level 3 less5 second responds time;

Level 4: less3 second responds time.

One round refers to complete one game which can determine whether the participants win or lose.

Notes: The participants can determine if they want to play level 3 or level 4 without forcing them up to level 3 or 4. During the intervention, 30 (100%) had ever played level 3 and 26 (87%) had ever played level 4.

| **Table S2. Demographic Information for participants in each group (N=56)** | | | |
| --- | --- | --- | --- |
| Data show mean ± standard deviation or number (%) | **Intervention**  (n=29) | **Control**  (n=27) | p-value |
| **Age** (years) | 66.45±5.23 | 68.07±5.78 | 0.274 |
| **Sex**  Female  Male | 19 (65.5%)  10 (34.5%) | 16 (59.3%)  11 (40.7%) | 0.783 |
| **Years in education** | 14.55±3.10 | 14.07±3.71 | 0.602 |
| **Employment status**  Full-time  Part-time  Homemaker  Retired | 4 (13.8%)  2 (6.9%)  3 (10.3%)  20 (69.0%) | 3 (11.1%)  1 (3.7%)  2 (7.4%)  21 (77.8%) | 0.890 |
| **Alcohol consumption**  Lifelong abstainer  Former drinker  Current drinker | 2 (6.9%)  12 (41.4%)  15 (51.7%) | 6 (22.2%)  9 (33.3%)  12 (44.4%) | 0.260 |
| **Tobacco smoking**  Never  Former smoker  Current smoker | 19 (65.6%)  9 (31.0%)  1 (3.4%) | 21 (77.8%)  6 (22.2%)  0 (0.0%) | 0.442 |
| **Immunization status**  Influenza  Pneumococcus  Varicella zoster  COVID-19 | 12 (41.4%)  5 (17.2%)  1 (3.4%)  25 (86.2%) | 15 (55.6%)  13 (48.1%)  3 (11.1%)  26 (96.3%) | 0.422  0.021  0.343  0.353 |
| **Medication usage**  Pharmaceutical agents  Health supplements | 22 (75.9%)  22 (75.9%) | 19 (70.4%)  19 (70.4%) | 0.765  0.765 |
| **Function assessment** |  |  |  |
| Cognitive performance (MoCA) | 27.45±2.32 | 26.63±2.34 | 0.195 |
| IPAQ (Kcal/week) | 2197.47±1882.48 | 1904.82±1102.20 | 0.485 |
| **Body composition** |  |  |  |
| Body Mass Index (kg/m^2^) | 24.26±3.28 | 24.09±2.71 | 0.838 |
| Skeletal muscle mass (kg) | 23.86±4.75 | 25.29±5.13 | 0.285 |
| Right arm muscle mass (kg) | 2.18±0.58 | 2.27±0.55 | 0.539 |
| Left arm muscle mass (kg) | 2.15±0.59 | 2.31±0.60 | 0.326 |
| Right leg muscle mass (kg) | 6.83±1.38 | 7.13±1.43 | 0.424 |
| Left leg muscle mass (kg) | 6.70±1.29 | 7.06±1.35 | 0.305 |
| Total body fat (%) | 30.07±7.14 | 28.29±7.27 | 0.361 |

**Table S3 Anatomical regions of structural and functional change between group**

|  | MNI coordinates | | | Cluster size | Maximum value | Anatomical region |
| --- | --- | --- | --- | --- | --- | --- |
|  | x | y | z |  |  |  |
| **GBV** | -48 | 40 | -16 | 269 | 3.82 | Lt. Frontal Pole |
|  | -13 | 25 | -25 | 182 | 3.42 | Lt. Frontal Orbital Cortex |
| **ReHo** | 36 | 8 | -19 | 57 | 4.05 | Rt. Insular Cortex |
| **fALFF** | -33 | 59 | -7 | 25 | 3.51 | Lt. Frontal Pole |

Peak of the group difference in structural and functional measures with a threshold of FWE-corrected *p*-value < 0.05.

Abbreviations: fALFF, fractional amplitude of low-frequency fluctuations; GMV, gray matter volume; Lt, left; MNI, Montreal Neurological Institute; ReHo, regional homogeneity; Rt, right.

**Table S4 Anatomical regions of intrinsic functional measures with delayed recall score**

|  | MNI coordinates | | | Cluster size | Maximum value | Anatomical region | |
| --- | --- | --- | --- | --- | --- | --- | --- |
|  | x | y | z |  |  |  |  |
| ***Positive*** | | | | | | |  |
| **ReHo** | 38 | -8 | -6 | 134 | 4.16 | Rt. Insular Cortex | |
| **fALFF** | -52 | 16 | 8 | 21 | 4.59 | Lt. Inferior Frontal Gyrus | |
|  | | | | | | |  |
| ***Negative*** | | | | | | |  |
| **ReHo** | 8 | 52 | 10 | 69 | 3.92 | Rt. Paracingulate Gyrus | |
|  | 36 | 48 | 36 | 74 | 3.66 | Rt. Frontal Pole | |
| **fALFF** | 4 | 44 | -10 | 72 | 6.08 | Rt. Paracingulate Gyrus | |

Peak of the relationship with intrinsic functional measures and delayed recall with a threshold of FWE-corrected *p*-value < 0.05.

Abbreviations: fALFF, fractional amplitude of low-frequency fluctuations; Lt, left; MNI, Montreal Neurological Institute; ReHo, regional homogeneity; Rt, right.

**Table S5** **Changes in MRI, cognitive performance, physical assessment, and bioassay findings (*N* = 56)**

| Variable | Intervention (*n* = 29) | | | | Control (*n* = 27) | | | | Intervention effect* | |
| --- | --- | --- | --- | --- | --- | --- | --- | --- | --- | --- |
|  | Baseline | 6 months | T1–T0 | *P* | Baseline | 6 months | T1–T0 | *P* | Estimate (95% CI) | *P* |
|  | Mean ± SD | |  |  | Mean ± SD | |  |  |  |  |
| Cognitive performance (MoCA) | | | | | | | | | | |
| Total score | 20.31 ± 9.23 | 28.31 ± 1.89 | 8.00 ± 9.70 | <.001 | 21.74 ± 12.93 | 26.81 ± 2.84 | 5.07 ± 13.15 | .06 | -0.676 (-1.659 to 0.306) | .17 |
| Delayed recall | 3.45 ± 1.35 | 4.28 ± 0.92 | 0.83 ± 1.54 | .007 | 3.70 ± 1.20 | 3.74 ± 1.43 | 0.037 ± 1.22 | .88 | -0.790 (-1.538 to -0.042) | .04 |
| Physical activity (IPAQ) | | | | | | | |  | | |
| Total physical activity  per week : METe (kcal per week) | 2146.32 ± 1788.72 | 2246.26 ± 1539.92 | 99.93 ± 11.06.38 | .63 | 1814.36 ± 1062.86 | 1885.38 ± 1400.47 | 71.02 ± 995.22 | .71 | -28.90 (-594.21 to 536.39) | .92 |
| Time spent sitting on weekdays (hours) | 155.86 ± 166.93 | 108.62 ± 145.74 | -47.23 ± 11  2.66 | .03 | 146.67 ± 200.61 | 171.11 ± 201.558 | 24.44 ± 147.66 | .48 | 71.67 (-7.76 to 141.11) | .08 |
| Resilience (BRS) | | | | | | | | | | |
| Average score | 3.97 ± 0.56 | 4.07 ± 0.46 | 0.10 ± 0.62 | .38 | 3.93 ± 0.68 | 4.00 ± 0.62 | 0.07 ± 0.55 | .49 | 0.007 (-0.282 to 0.296) | .96 |
| Demoralization (DS-MV) | | | | | | | | | | |
| Total score | 20.31 ± 9.23 | 17.76 ± 9.74 | -2.55 ± 10.02 | .18 | 21.74 ± 12.93 | 21.56 ± 10.55 | -0.19 ± 7.42 | .90 | 2.366 (-2.387 to 7.120) | .32 |
| Quality of life (EQ-5D) | | | | | | | | | | |
| Visual analog score | 78.83 ± 9.25 | 80.55 ± 10.69 | 1.72 ± 9.96 | .36 | 78.04 ± 9.06 | 75.74 ± 9.27 | -2.30 ± 8.16 | .16 | -4.038 (-8.941 to 0.863) | .10 |
| Body composition and physical performance | | | | | | | | | | |
| Height (cm) | 160.79 ± 6.59 | 160.79 ± 6.62 | 0.03 ± 0.63 | .77 | 162.48 ± 1.47 | 162.26 ± 1.43 | -0.22 ± 0.75 | .14 | -0.265 (-0.528 to -0.003) | .048 |
| Diastolic BP (mmHg) | 80.24 ± 11.86 | 77.31 ± 9.46 | -2.93 ± 7.18 | .04 | 76.37 ± 10.96 | 76.81 ± 9.62 | 0.44 ± 5.32 | .67 | 3.501 (0.091 to 6.911) | .04 |
| Right leg muscle mass (kg) | 6.85 ± 1.45 | 6.62 ± 1.46 | -0.22 ± 0.50 | .02 | 7.10 ± 1.51 | 6.92 ± 1.54 | -0.18 ± 0.37 | .02 | -0.080 (-0.246 to 0.084) | .33 |
| Left leg muscle mass (kg) | 6.66 ± 1.27 | 6.58 ± 1.37 | -0.08 ± 0.40 | .27 | 7.08 ± 1.44 | 6.85 ± 1.43 | -0.23 ± 0.44 | .01 | -0.065 (-0.218 to 0.088) | .40 |
| Limbs total muscle mass | 23.90 ± 4.78 | 23.69 ± 4.92 | -0.20 ± 1.05 | .30 | 25.26 ± 5.09 | 14.70 ± 4.52 | -0.56 ± 2.47 | .25 | -0.545 (-1.513 to 0.421) | .26 |
| Appendicular skeletal muscle mass (kg/m^2^) | 6.83 ± 1.10 | 6.72 ± 1.13 | -0.10 ± 0.41 | .18 | 7.04 ± 1.29 | 6.85 ± 1.13 | -0.19 ± 0.48 | .06 | 0.028 (-0.091 to 0.148) | .64 |
| Total body fat percentage | 19.14 ± 6.26 | 18.62 ± 5.49 | -0.52 ± 3.73 | .46 | 18.00 ± 5.65 | 17.96 ± 5.36 | -0.04 ± 2.73 | .95 | 0.373 (-1.334 to 2.079) | .66 |
| Phase angle (rad) | 5.17 ± 0.45 | 5.29 ± 0.75 | 0.12 ± 0.73 | .40 | 5.16 ± 0.46 | 5.15 ± 0.46 | -0.01 ± 0.33 | .91 | -0.185 (-0.407 to 0.035) | .10 |
| Hematology and urinalysis | | | | | | | | | | |
| Cholesterol (mg/dL) | 195.03 ± 40.28 | 190.07 ± 41.04 | -4.97 ± 30.71 | .39 | 187.41 ± 31.52 | 199.26 ± 34.33 | 11.85 ± 34.24 | .08 | 16.817 (-0.587 to 34.221) | .06 |
| Thyrotropin (uIU/mL) | 2.10 ± 1.20 | 1.88 ± 0.85 | -0.23 ± 0.78 | .13 | 1.87 ± 1.22 | 2.19 ± 1.31 | 0.32 ± 0.98 | .10 | -0.545 (-1.018 to -0.072) | .02 |
| SD, standard deviation; CI, confidence interval; TIV, total intracranial volume; MoCA, Montreal Cognitive Assessment; IPAQ, International Physical Activity Questionnaire; BRS, Brief Resilience Scale; EQ-5D, EuroQol Five-Dimensional; DS-MV, Demoralization Scale-Mandarin Version.  ^*^Intervention effect computed as the between-group difference in mean change from baseline to month 6 (change in intervention group minus change in control group). Intervention effect after adjustment for age and sex except for gray matter volume alteration. Gray matter volume alteration after adjustment for age, sex, and TIV. | | | | | | | | | | |

**Table S6 Mahjong performance and secondary outcomes**

|  | Unstandardized | | Standardized | t | p-value |
| --- | --- | --- | --- | --- | --- |
|  | β | SE | β |  |  |
| **Affecting decreased blood cholesterol** | | | | | |
| Average time per playing -0.68 0.30 -0.40 -2.28 .031 | | | | | |
| **Affecting increased blood high-density lipoprotein** | | | | | |
| Numbers of 20 second responds time practice sessions | 0.07 | 0.04 | 0.37 | 2.08 | .047 |
| **Affecting increased blood thyrotropin** | | | | | |
| 7th month walking steps | -0.00 | 0.00 | -0.41 | 2.31 | .029 |
| SE, standard error. | | | | | |
