# Supplementary material for: Digital Mahjong-Based Cognitive Engagement Intervention for Older Adults: Randomized Controlled Trial
Source: JMIR Aging. 2026 Jul 23;9:e85339. doi: 10.2196/85339 (PMC13448193; doi:10.2196/85339)
Supplement: Supplementary file 4 [file aging_v9i1e85339_app3.docx]

**Multimedia Appendix 3. Sensitivity analysis.**

In our study, we performed a sensitivity analysis to determine whether our MRI findings were driven by MRI delay duration rather than the intervention. Therefore, we re-analyzed the MRI image features by including the "duration of MRI delay" as an additional covariate in our ANCOVA models. In the between-group comparisons, the results for fALFF and GMV remained largely consistent with our original findings. However, for the ReHo analysis, we observed that including the MRI delay duration as a covariate reduced the statistical power of the ANCOVA model. Although the original insular activation (in the intervention group) did not survive the strict cluster-level FWE correction in this controlled model, the pattern of the ReHo activation remained consistent. When we examined the data at a relaxed threshold (uncorrected p < 0.05, cluster size > 100 voxels), the ReHo activation of the insula was still clearly identifiable, suggesting that the physiological effect remains present but fell just below the strict significance threshold due to the reduced degrees of freedom.

In contrast, the brain-behavior correlation analyses demonstrated remarkable stability. The positive association between improved delayed recall and increased ReHo in the right insula after controlling for the MRI delay, with the peak coordinates remaining identical to the original analysis. More importantly, we also used partial Pearson correlation analysis, accounting for age, sex and MRI delay to estimate the associations between brain signature changes and response practice sessions. The results were consistent with our primary findings, confirming a significant correlation between the right insula and practice intensity. These results supported robust evidence that the intervention-induced neuroplasticity mechanism is reliable and was not confounded by the delayed duration.

**Table S1. Sensitivity analysis: Anatomical regions of structural and functional change between group**

|  | MNI coordinates | | | Cluster size | Maximum value | Anatomical region |
| --- | --- | --- | --- | --- | --- | --- |
|  | x | y | z |  |  |  |
| **GBV** | -48 | 40 | -16 | 316 | 3.98 | Lt. Frontal Pole |
| **ReHo** | 38 | 64 | --2 | 77 | 3.58 | Rt. Frontal Pole |
| **fALFF** | -33 | 59 | -5 | 25 | 3.75 | Lt. Frontal Pole |

Peak of the group difference in structural and functional measures with a threshold of FWE-corrected *p*-value < 0.05.

Abbreviations: fALFF, fractional amplitude of low-frequency fluctuations; GMV, gray matter volume; Lt, left; MNI, Montreal Neurological Institute; ReHo, regional homogeneity; Rt, right.

**Table S2. Sensitivity analysis: Anatomical regions of intrinsic functional measures with delayed recall score**

|  | MNI coordinates | | | Cluster size | Maximum value | Anatomical region |
| --- | --- | --- | --- | --- | --- | --- |
|  | x | y | z |  |  |  |
| ***Positive*** | | | | | | |
| **ReHo** | 38 | -8 | -6 | 74 | 3.81 | Rt. Insular Cortex |
| **fALFF** | -50 | 14 | 7 | 25 | 4.18 | Lt. Inferior Frontal Gyrus |
|  | | | | | | |
| ***Negative*** | | | | | | |
| **ReHo** | 8 | 52 | 10 | 71 | 4.28 | Lt. Superior Frontal Gyrus |
|  | -2 | 46 | 38 | 59 | 3.86 | Lt. Superior Frontal Gyrus |
|  | 36 | 48 | 36 | 92 | 3.68 | Rt. Frontal Pole |
| **fALFF** | 4 | 44 | -10 | 66 | 6.16 | Rt. Paracingulate Gyrus |
|  | 0 | 46 | 40 | 27 | 4.66 | Rt. Superior Frontal Gyrus |
|  | 20 | 65 | 23 | 26 | 4.31 | Rt. Frontal Pole |

Peak of the relationship with intrinsic functional measures and delayed recall with a threshold of FWE-corrected *p*-value < 0.05.

Abbreviations: fALFF, fractional amplitude of low-frequency fluctuations; Lt, left; MNI, Montreal Neurological Institute; ReHo, regional homogeneity; Rt, right.

**Figure S1. Sensitivity analysis: Brain measure changes between control group and intervention group**

Hot (red-yellow) regions reflect significantly increased change in intervention group. Cold (blue-green) regions reflect significantly decreased change in intervention group. Abbreviations: fALFF, fractional amplitude of low-frequency fluctuations; FWE, family-wise error; GMV, gray matter volume; ReHo, regional homogeneity.

**Figure S2. Sensitivity analysis: Relationship between brain image measures and intervention effect cognition outcome**

Multiple linear regression analyses examining the association between brain measures and cognitive outcomes were adjusted for age, sex, and the duration of MRI delay. Hot (red-yellow) regions reflect significantly increased change in intervention group. Cold (blue-green) regions reflect significantly decreased change in intervention group. Abbreviations: fALFF, fractional amplitude of low-frequency fluctuations; FWE, family-wise error; ReHo, regional homogeneity.

**Figure S3. Sensitivity analysis: Associations between brain signature changes and response practice session**

Relationships were estimated using partial Pearson correlation analysis, accounting for age, sex and MRI delay duration. Significant correlations were marked with a black box (FDR-corrected P < .05). Abbreviations: fALFF, fractional amplitude of low-frequency fluctuations; FDR, false discovery rate; Lt, Left; ReHo, regional homogeneity; Rt, right.
